# Enhanced tenacity of mycobacterial aerosols from necrotic neutrophils

**DOI:** 10.1038/s41598-020-65781-9

**Published:** 2020-06-08

**Authors:** E. Pfrommer, C. Dreier, G. Gabriel, T. Dallenga, R. Reimer, K. Schepanski, R. Scherließ, U. E. Schaible, T. Gutsmann

**Affiliations:** 1 Heinrich Pette Institute, Leibniz Institute for Experimental Virology, Hamburg, 20251 Germany; 2 Forschungszentrum Borstel - Leibniz Lung Center, Borstel, 23845 Germany; 3 Leibniz Institute for Tropospheric Research, Leipzig, 04318 Germany; 4 Leibniz Research Alliance INFECTIONS’21, Borstel, 23845 Germany; 5 German Center for Infection Research (DZIF), Partner Site Hamburg-Lübeck-Borstel, Germany; 6 Christian Albrechts University of Kiel, 24118 Kiel, Germany

**Keywords:** Pathogens, Pathogens, Tuberculosis, Tuberculosis

## Abstract

The tuberculosis agent *Mycobacterium tuberculosis* is primarily transmitted through air, but little is known about the tenacity of mycobacterium-containing aerosols derived from either suspensions or infected neutrophils. Analysis of mycobacterial aerosol particles generated from bacterial suspensions revealed an average aerodynamic diameter and mass density that may allow distant airborne transmission. The volume and mass of mycobacterial aerosol particles increased with elevated relative humidity. To more closely mimic aerosol formation that occurs in active TB patients, aerosols from mycobacterium-infected neutrophils were analysed. Mycobacterium-infected intact neutrophils showed a smaller particle size distribution and lower viability than free mycobacteria. In contrast, mycobacterium-infected necrotic neutrophils, predominant in *M. tuberculosis* infection, revealed particle sizes and viability rates similar to those found for free mycobacteria, but in addition, larger aggregates of viable mycobacteria were observed. Therefore, mycobacteria are shielded from environmental stresses in multibacillary aggregates generated from necrotic neutrophils, which allows improved tenacity but emphasizes short distance transmission between close contacts.

## Introduction

At a global level, tuberculosis (TB) continues to be a major cause of morbidity and mortality, causing 1.2 million deaths in 2018^1^. With more than 160,000 new cases in 2017, the spread of drug-resistant TB highlights the importance of understanding the transmission properties of its agent, *Mycobacterium tuberculosis* (*M. tuberculosis*)^1^. An active TB patient can infect up to 15 sentinel hosts annually when unrecognized^2^. Transmission predominantly occurs via the airborne route^3–5^. Successful airborne transmission is influenced mainly by four factors: (I) the pathogen-induced pathology, which is based on mycobacterial virulence and host response-associated inflammatory processes promoting necrotic cell death, exacerbated pathology and expelling of the bacteria as aerosol droplets by coughing^6^; (II) the aerobiology of pathogen-containing aerosols, which facilitates airborne transmission as determined by size and other physicochemical characteristics of the aerosol particles^7^; (III) the resilience of the pathogen to environmental influences^8^; and (IV) the availability and susceptibility of a new potential host. These factors determine the retention period and viability of airborne mycobacteria, described as tenacity. Here, we focus mainly on factor II, particularly on the influence of intact or necrotic neutrophils on the particle size and viability of *in vitro*-generated mycobacterium-containing aerosols. Tuberculosis can transmit over both long and short distances. In this paper, we mimic the close contact situation and focus on short-distance transmission. For that, we kept environmental conditions constant in the transmission experiments shown.

A cycle of events leads to airborne human-to-human *M. tuberculosis* transmission. This cycle is initiated when a *M. tuberculosis-*containing water droplet is inhaled and deposited onto the alveolar surface, where it is engulfed by an alveolar macrophage for subsequent transport into the interstitium. Following the onset of acquired immunity, granulomas restrict bacterial growth and spread but serve as a source for reactivation^9,10^. Upon reactivation, hallmarks of active TB include necrotizing granulomas, exacerbating pathogenesis and neutrophil influx^11^. Increasing evidence has emerged that neutrophils play an important role in exacerbating lung pathology in active TB, facilitating mycobacterial transmission by cough-expelled aerosols^12–14^. Virulent *M. tuberculosis* quickly induces necrotic cell death in neutrophils upon infection in a reactive oxygen intermediate (ROI)-dependent manner, which is not seen upon infection with attenuated strains lacking the entire virulence-associated RD1 region, such as *Mycobacterium bovis* Bacillus Calmette Guerin (BCG)^11,14^. *M. tuberculosis* can initiate necrosis, particularly in neutrophils, which facilitates access to alveolar or bronchiolar airways where it can form biofilms, so-called necrosis-associated extracellular clusters^15^, which allow airborne transmission^11^. Although neutrophils were the most abundant infected cells in sputum and broncho-alveolar lavage (BAL) fluid from TB patients^13^, the role of necrotic neutrophil mycobacterial aerosol properties and their influence on mycobacterial tenacity, i.e., their viability and residency in environmental air, is still unknown.

Infectious *M. tuberculosis* bioaerosols may exist as single bacterial cells, as multibacillary aggregates or as mycobacteria carried by host-derived materials^6,16,17^. Most infectious particles generated from the human respiratory tract occur primarily as droplet nuclei with a diameter of 0.5–12 µm^17^. The range of particle sizes in an infectious aerosol *in vivo* depends on a number of factors, including but not limited to the mechanism of aerosol generation and the liquid content and viscosity of the aerosolized fluid^6^. In the context of infectious disease transmission, many processes involving the respiratory tract, mainly coughing and sneezing but also singing and talking, can generate aerosols carrying pathogens in conjunction with body fluids^16,18,19^. Talking for 5 min can generate approximately 300 aerosol nuclei, whereas a similar number is expelled by just one cough. The deposition of airborne particles in the human respiratory tract is defined by a wide spectrum of physical characteristics of the particle and physiological factors. Usually, ultrafine particles (aerodynamic diameter: <0.1 µm) and intermediate particles (aerodynamic diameter: 0.1–3 µm) are able to penetrate the deep lung regions, where they may finally settle in the alveoli^6,7,20,21^. Large particles (aerodynamic diameter: >3–5 µm) are deposited mainly in the extra-thoracic part of the respiratory tract^22^. To simulate disposition in the human respiratory tract, an Andersen cascade impinger can be used, as it shows a comparable distribution of airborne particles on different levels of the impinger^23^.

By definition, aerosols are suspensions of solid or liquid particles in air that are small enough that they remain airborne for a prolonged period of time^7,24^. The fate of an aerosol in the environment is governed by physical processes^7^. The settling velocity of airborne particles is influenced by gravity and directly correlates with (I) the particle’s aerodynamic size, as larger particles settle more rapidly than smaller particles, and (II) its mass density, as denser particles will fall out of the air more quickly^7^. After emission of *M. tuberculosis*-containing aerosols from the respiratory tract, they encounter environmental factors, including relative humidity (RH) and UV radiation. RH has long been recognized as a key factor in bioaerosol activity. Peccia *et al*. (2004) conducted decay experiments in an aerosol reactor (0.8 m^3^) to determine the impact of RH and UV radiation on airborne bacteria and showed that a high RH is protective against UV radiation for airborne bacteria, such as BCG^25^. Depending on the RH of the environment, water layers start to evaporate and condense around the bacterium^6,26^. Biological particles can also take up water, leading to hygroscopic growth. Thus, the RH of an indoor environment can have a dramatic effect on the particle’s tenacity, as it affects the aerodynamic size, the length of its airborne time and the viability of the bacteria^4,27,28^. For TB, it was proposed that an elevated RH will increase the transmission rate of *M. tuberculosis*^26,27^.

To understand the airborne transmission of respiratory infectious diseases, it is necessary to understand the biophysical characteristics of mycobacterial aerosol transmission. This understanding will allow us to assess risks of infection and to design protocols to better protect at-risk groups, such as health workers or household contacts, and to ultimately develop prevention measures for pathogen shedding.

We found that the mass density and aerodynamic size of mycobacterium-containing aerosols would allow long-distance transport through the air, but the viability of airborne mycobacteria is quickly reduced. However, aerosol particles derived from necrotic neutrophils contained increased numbers of viable bacteria, particularly in large aggregates. We therefore postulate that multibacillary clumps, though they primarily allow short distance transport, are a major risk factor for mycobacterial transmission, as they deliver increased numbers of viable mycobacteria, probably due to improved protection from environmental influences. This process can be promoted by necrotic neutrophil cell death associated with active TB.

## Results

### Influence of aldehyde fixation on the biophysical properties of mycobacteria

*M. tuberculosis* is an obligate human safety class 3 pathogen and therefore can be handled only in a BSL-3 facility. As most of our aerosol generating and analysing infrastructures are located in BSL-2 facilities, the samples required inactivation using aldehyde fixation before leaving the BSL-3 laboratory for analyses. Examining the influence of different protocols for aldehyde fixations, we observed that inactivation with glutaraldehyde (GA) or paraformaldehyde (PFA) increased the volume of BCG compared to that of native BCG, as determined by atomic force microscopy (Fig. S1a). In contrast, only slight size differences were measured in water solution between GA- or PFA-fixed and non-fixed mycobacteria with dynamic light scattering (Fig. S1b). These results suggest altered water exchange across the mycobacterial cell envelope due to aldehyde fixation. To be able to experiment with live and native mycobacteria, the observed fixation effect on mycobacterial cellular volume prompted us to work with the attenuated vaccine strain BCG, a structurally and genetically close relative of *M. tuberculosis*.

### Mass density and hygroscopicity of mycobacteria

**Figure 1:**
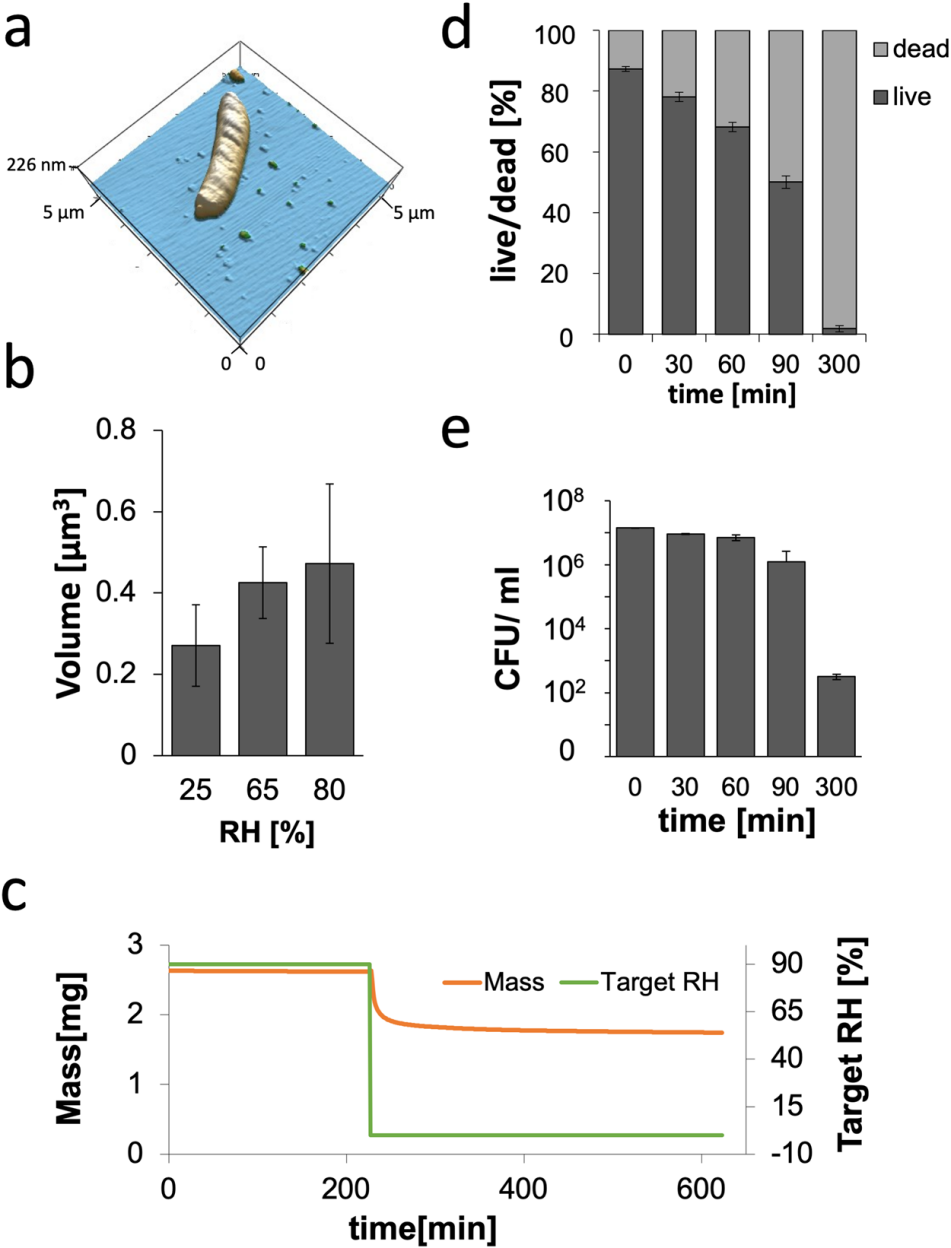
Biophysical properties of mycobacteria: (**a**) Representative 3D reconstruction of a mycobacterial cell using Igor Pro software to calculate the volume of a single mycobacterium in air. (**b**) Volume of a single mycobacterium in air under different RH. The RH was adjusted by deliquescence salts inside a closed fluid chamber (n = 3 per humidity, p = 0.0008). **(c)** Gravimetric measurement of changes in the mass of mycobacterial pellets upon a decrease in the RH from 90% to 0% RH at 25 °C (n = 2). **(d**,**e)** Ability of soluble mycobacteria from broth cultures to survive for different periods of drying. **(d**) Live/dead staining using a LIVE/DEAD BacLight Bacterial Viability Kit (Invitrogen), depicted as the ratios of live vs. dead mycobacteria out of 100 mycobacteria analysed (n = 3). **(e)** Independent determination of CFU counts (n = 3).

The ability of aerosol particles to remain airborne strongly depends on biophysical properties, including mass density and hygroscopicity. Mass density was determined indirectly by calculating the volume of single bacterial cells at 65% RH based on AFM measurements and 3D reconstructions (Fig. 1a) and was found to be (0.43 ± 0.08) µm^3^. The weight of a single mycobacterium was determined to be 7.66 * 10^−15^ ± 6.92 * 10^−16^ g. The mass density of one mycobacterium cell was therefore calculated to be 1.79 ± 0.08 g/cm^3^.

The hygroscopicity of mycobacteria, i.e., their ability to take up water from the environment, was analysed by monitoring changes in volume or mass of either a single bacterium as measured by AFM or a bacterial pellet by gravimetry, respectively (Fig. 1b and Fig. 1c). At 25% RH, the average volume of a single mycobacterial cell was 0.26 ± 0.09 µm^3^, which almost doubled to 0.47 ± 0.19 µm^3^ at 80% RH. At 65% RH, the average volume was 0.43 ± 0.08 µm^3^, suggesting a stage of saturation between 65 and 80% RH, in which the mycobacterium was not able to take up additional water (Fig. 1b). Regarding the mycobacterial mass, a loss of 30% was observed when RH was reduced from 90% to 0% (Fig. 1c).

### Resilience of mycobacteria to air

As mycobacterial aerosols face the possibility of dying from dehydration while travelling through air, their ability to maintain viability was analysed by determining the ratios between live and dead mycobacteria and CFU analysis as a measure of mycobacterial growth on plates at different time points after exposure to ambient air at room temperature (20–25 °C) and a RH between 60–70%. In the original solution of mycobacteria grown in broth and used for the experiments, 12.73 ± 0.74% were not viable. Due to dehydration, the number of dead bacteria gradually increased from 12.73 ± 0.74% to 21.95 ± 1.53% after 30 min, to 31.8 ± 1.51% after 60 min, to 49.96 ± 2.02% after 90 min, and finally to 98.23 ± 0.98% after 300 min (Fig. 1d). Accordingly, the viability of mycobacteria measured by CFU decreased from 1.41 10^7^ ± 2.15 10^5^ by 34.5% (9.23 10^6^ ± 2.87 10^5^);and 50.9% (1.21 10^6^ ± 1.45 10^5^) after 30 min and 90 min in air, respectively, but became further reduced by 99.9% (3.2 10^2^ ± 317) after 300 min (Fig. 1e).

### Aerodynamic size and survival rates of airborne mycobacteria

**Figure 2:**
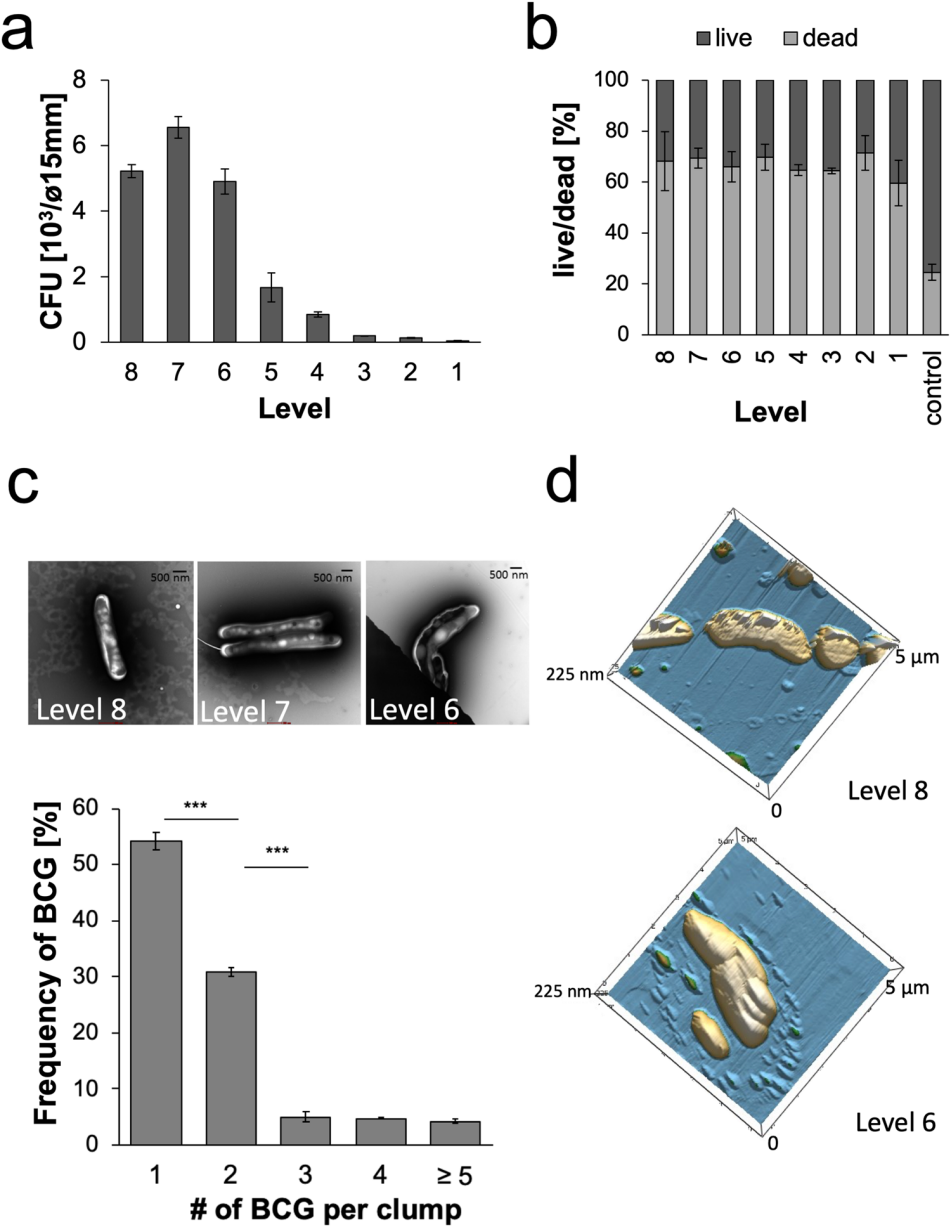
Properties of mycobacterium-containing aerosols: (**a**) CFU of mycobacteria collected from the different levels of the Andersen impinger upon aerosolization in PBS at a 28 l/min airflow (n = 5; p > 0.001). (**b**) Percentages of live vs. dead mycobacteria as determined by live/dead staining upon aerosolization in PBS assessed immediately after settling for 10 min at the different impinger levels in comparison to control samples directly transferred from culture into PBS for viability staining before nebulization (n = 3, out of 100 mycobacteria, p > 0.0015). **(c)** Frequency of different mycobacterial cell numbers within aerosol particles, as depicted by electron microscopy analysis counted on levels 1–8 (n = 3, p > 0.0001). **(d)** Representative 3D reconstruction of airborne mycobacteria collected at different levels and analysis by AFM (the mean and standard deviation of three independent experiments are shown).

Successful airborne transmission of mycobacteria depends on their aerodynamic size and ability to survive environmental conditions in the air. An Andersen impinger allows simultaneous assessment of both aerodynamic size and airborne pathogen viability as measured by CFU recovery and live/dead staining as an assessment of membrane damage. Depending on their velocity, particles with smaller aerodynamic diameters settle lower, and those with larger aerodynamic diameters settle at higher levels. During the sampling time of 10 min, aerosols were continuously generated by the jet nebulizer. Viability testing by CFU of the aerosol collection device (ACD) ambient air samples from the Andersen impinger revealed that 85% of all viable mycobacteria were found on levels 6–8, corresponding to an aerodynamic diameter between 0.22 and 1.7 µm (Fig. 2a, Table 1). Live/dead staining showed a significant loss in the viability of airborne BCG when compared to that of control non-airborne mycobacteria in PBS (Fig. 2b). Notably, as determined by live/dead staining, transfer into PBS already affected mycobacterial membrane integrity, as 24.56 ± 3.1 out of 100 mycobacterial cells were already dead before aerosolization (compare the control in Fig. 2b to timepoint 0 in Fig. 1d).

**Table 1:** Relative distribution of aerosol sizes: Each level of the Andersen impinger correlates with a diameter cut off [μm] given by the manufacturer.

Level	1	2	3	4	5	6	7	8
Diameter cut off [µm]	8.0	6.5	5.2	3.5	2.6	1.7	1.0	0.22
Rel. distr. of mycobacteria [%]	0.4	0.8	1.4	2.9	9.4	27.3	40.6	17.1
Std. deviation	0.3	0.3	0.8	2.0	3.3	1.7	4.3	3.9

The relative distribution of the mycobacteria was determined by electron microscopy (3 independent experiments with 100 bacterial cells assessed in each one).

Sampling the different levels of the Andersen impinger for EM analysis revealed 53 ± 2% of mycobacterial particles as singlets, 32 ± 1% as doublets and a small number as clumps of more than 5 bacteria (Fig. 2c). The aerodynamic diameter of mycobacterium-containing particles was linked to the number of mycobacteria, as singlets were found mainly on levels 7 and 8 and doublets primarily on levels 6 and 7. Clumps, however, of more than 5 mycobacteria were observed predominantly on levels 1 and 2. AFM analysis of unfixed mycobacterial aerosol particles further confirmed that most particles represented single cells, whereas only a small fraction of those were found as large clumps (Fig. 2d).

**Figure 3:**
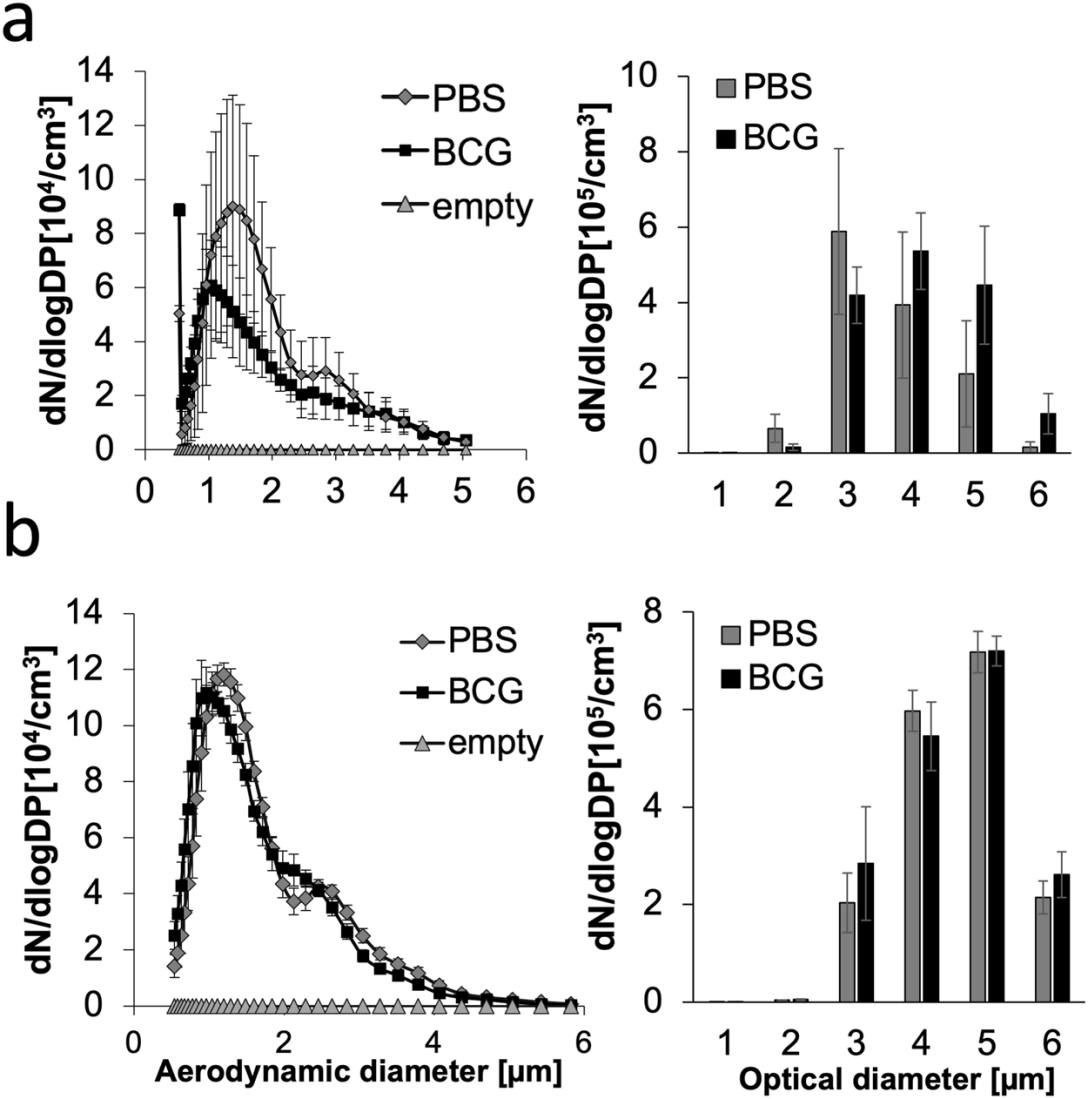
Aerodynamic and optical diameters of empty PBS aerosols and mycobacterium-containing aerosol particles determined by and aerodynamic particle spectrometer (APS): (**a**) Left: Distribution of aerodynamic diameters of jet-nebulized buffer and mycobacterium-containing aerosols (OD 0.6, 580 nm). Right: Distribution of optical diameters of jet-nebulized buffer and mycobacterium-containing aerosols (n = 3). **(b)** Left: Frequency of aerosol particles with a certain aerodynamic diameter upon ultrawave nebulization. Right: Optical diameters of ultrawave-nebulized buffer and mycobacterium-containing aerosols (n = 3).

To further confirm the aerodynamic diameter of mycobacterium-containing aerosols determined by the Anderson impinger, we additionally measured the flight time of aerosol particles in a fixed airflow of 1 l/min using an aerosol particle spectrometer (APS). Simultaneously, the optical diameter of each particle was determined. The main aerodynamic diameter was almost twice as high in empty PBS vs. mycobacterium-containing aerosols when aerosols were generated by jet nebulization but not in a statistically significant manner (p = 0.292) (Fig. 3a left). Mycobacterium-containing aerosols had aerodynamic diameters varying between 0.5 and 5 µm, with most aerosols showing an aerodynamic diameter of 0.89 µm, whereas the main diameter of empty PBS aerosols was 1.38 µm. In contrast to the aerodynamic diameters, the optical diameters of mycobacterium-containing aerosols generated by jet nebulization were larger than those of empty PBS aerosols but not statistically significantly different (p = 0.608) (Fig. 3a right). Aerosols generated by an ultrawave (Piezo) nebulizer show reduced variability in the aerodynamic diameters measured (compare Fig. 3a left and 3b left). Additionally, no difference was measured in the optical diameters between empty PBS and mycobacterium-containing aerosols (Fig. 3 left).

**Figure 4:**
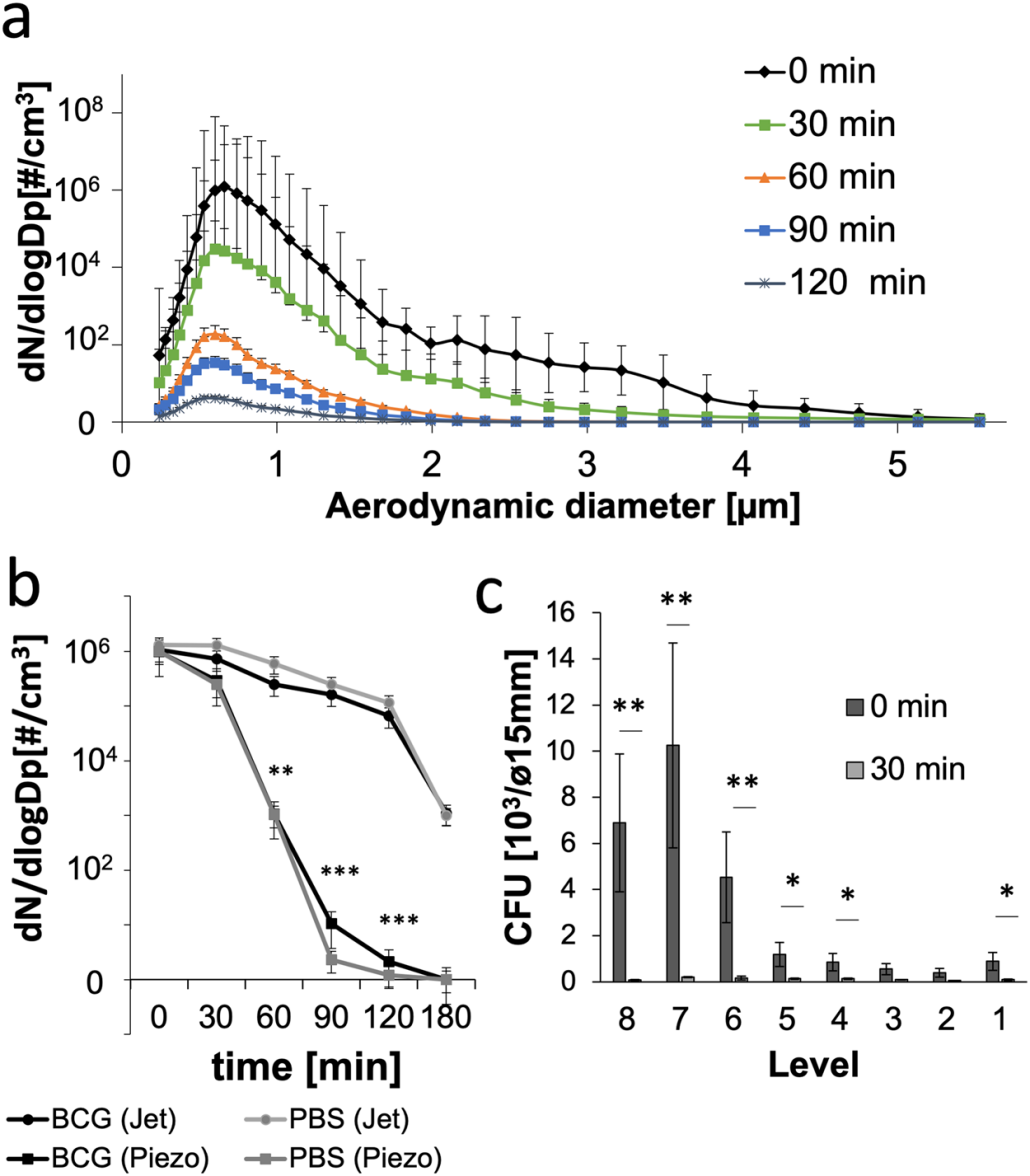
Tenacity of mycobacterium-containing aerosols. (**a**) Distribution of aerodynamic diameters of aerosols generated by jet nebulization from either buffer (PBS) or mycobacterial suspensions in PBS in ACD air at different time points after aerosol generation (n = 3). (**b**) Settling time of jet-nebulized aerosols vs ultrawave-nebulized aerosols (n = 3, **p > 0.001, ***p > 0.0001). (**c**) CFU of airborne mycobacteria collected from the different levels of an Andersen impinger at the starting time point of jet nebulization and 30 min later (n = 3, *p > 0.01, **p > 0.001).

Although aerodynamic diameter distributions at different time points after nebulization did not differ, we hypothesize that particles of larger aerosol sizes may drop out of air earlier than smaller particles. Therefore, we analysed the tenacity of mycobacterial aerosols, as represented by their viability and determined by recovered CFU and residency in the aerosol phase. Using the jet nebulizer, the total numbers of detectable aerosol particles became drastically reduced 60 min after nebulization initiation (Fig. 4a). Similar results were obtained using the ultrawave nebulizer (results not shown). Although both nebulizer systems generated comparable numbers of aerosol particles of similar aerodynamic diameters, those generated by ultrawave nebulization were lost faster from ACD air than those from the jet nebulization (Fig. 4b). Additionally, those aerosols contained reduced numbers of viable mycobacteria (Fig. S2). Interestingly, the large number of aerosol particles detected by APS 30 min after jet nebulization no longer contained viable mycobacteria when assessed by CFU (Fig. 4c).

### The role of neutrophil necrosis in the formation of mycobacterium-containing aerosols

**Figure 5:**
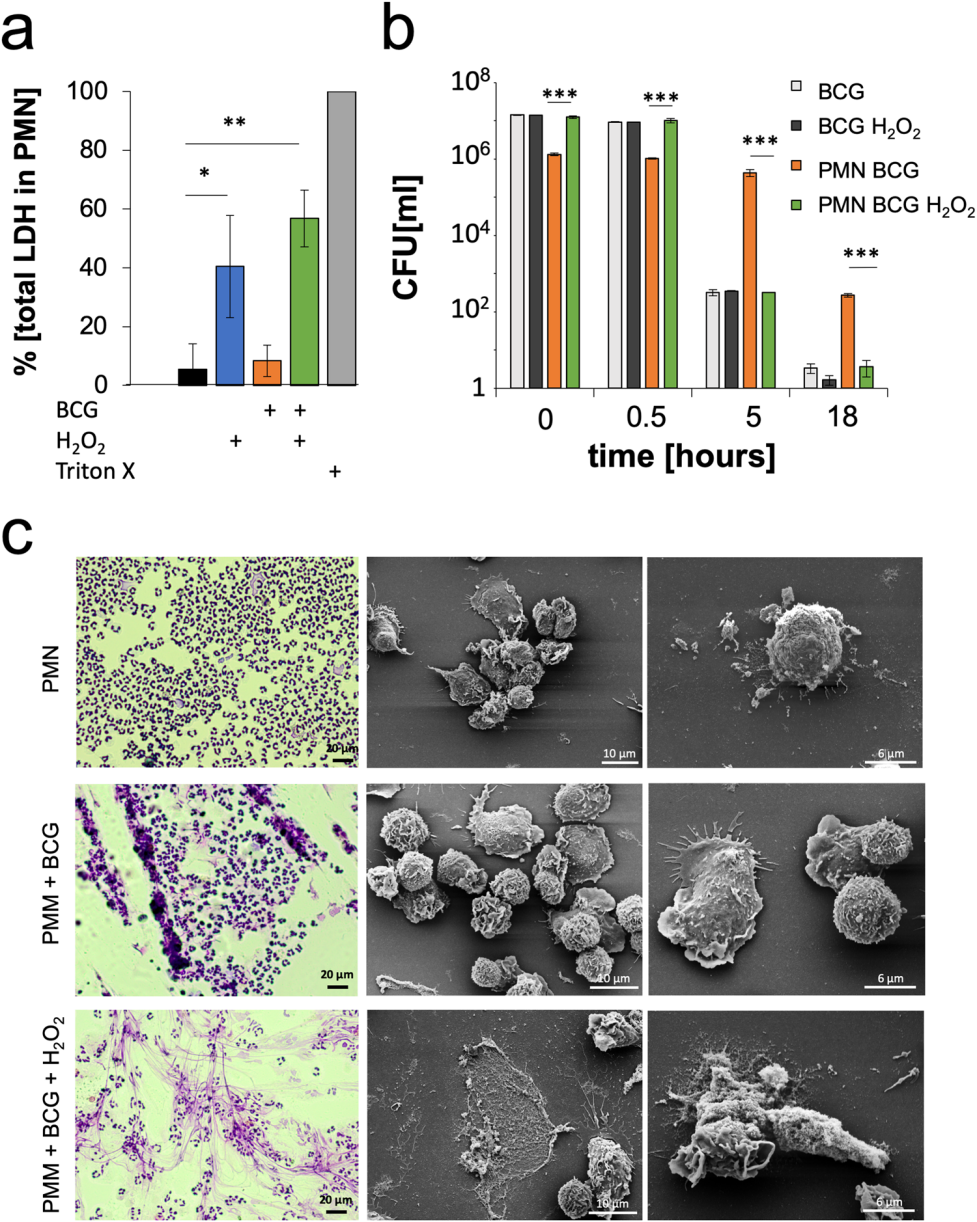
Mimicking mycobacterial-infected neutrophil necrosis that occurs in active TB patients. (**a**) Human neutrophils isolated from peripheral blood were infected with *M. bovis* BCG and either left untreated or treated with H_2_O_2_ to induce necrotic cell death. Necrotic cell death was assessed by measuring the activity of the cytoplasmic enzyme LDH in culture supernatants (n = 3 *p > 0.01**p > 0.001). (**b**) Viability assessed by CFU of mycobacteria recovered from *M. bovis* BCG-infected neutrophils that were either treated with H_2_O_2_ to induce necrotic cell death or left untreated and succumbed to default apoptotic cell death. CFU were determined upon exposure to ambient air for the indicated time periods (n = 3, **p > 0.001, ***p > 0.0001). (**c**) Morphological analysis of human neutrophils either left uninfected or infected with *M. bovis* BCG without or with H_2_O_2_ treatment by H&E staining and SEM (n = 3).

To mimic reactive oxygen species (ROS)-induced necrotic cell death in BCG-infected neutrophils as described for virulent *M. tuberculosis* but not attenuated mycobacteria, we used H_2_O_2_ to induce necrotic cell death in neutrophils. Untreated neutrophils or those infected with BCG showed a necrosis rate of <10 ± 8.6%. H_2_O_2_ treatment of non-infected neutrophils by itself induced a necrosis rate of >40 ± 17.4%. The necrosis rate was further increased to >56 ± 9.6% when the neutrophils were previously infected with BCG (Fig. 5a).

To study whether the passage of mycobacteria through neutrophils affects their viability upon exposure to air, we analysed mycobacterial CFUs from samples containing either free mycobacteria or mycobacteria within infected neutrophils, which were either untreated or exposed to H_2_O_2_ to induce necrotic cell death. The samples were applied directly to the slides. Under all three conditions, mycobacterial loads were comparable, with only a slight reduction in viability 0.5 h after exposure to ambient air. After 5 h of exposure to ambient air, mycobacterial viability was not as drastically reduced in intact neutrophils compared to that of mycobacteria from H_2_O_2_-treated necrotic neutrophils or free mycobacteria without H_2_O_2_ treatment. Even after 18 h of exposure to ambient air, 269 ± 27 mycobacterial CFUs were still detectable when they were shielded in intact neutrophils. However, only 3.67 ± 0.5 CFUs were recovered from mycobacteria in H_2_O_2_-treated neutrophils or free neutrophils with or without H_2_O_2_ treatment (Fig. 5b).

H_2_O_2_-induced necrotic cell death of BCG-infected neutrophils was also visualized by light microscopy and SEM (Fig. 5c). Untreated neutrophils isolated from human peripheral blood showed a typical round morphology and segmented nuclei. Infection with BCG reduced the number of neutrophils and enhanced aggregation. Treatment of BCG-infected neutrophils with H_2_O_2_ led to neutrophil cell membrane disintegration and filamentous cell remnants, as described previously^28^ (Fig. 5c).

**Figure 6:**
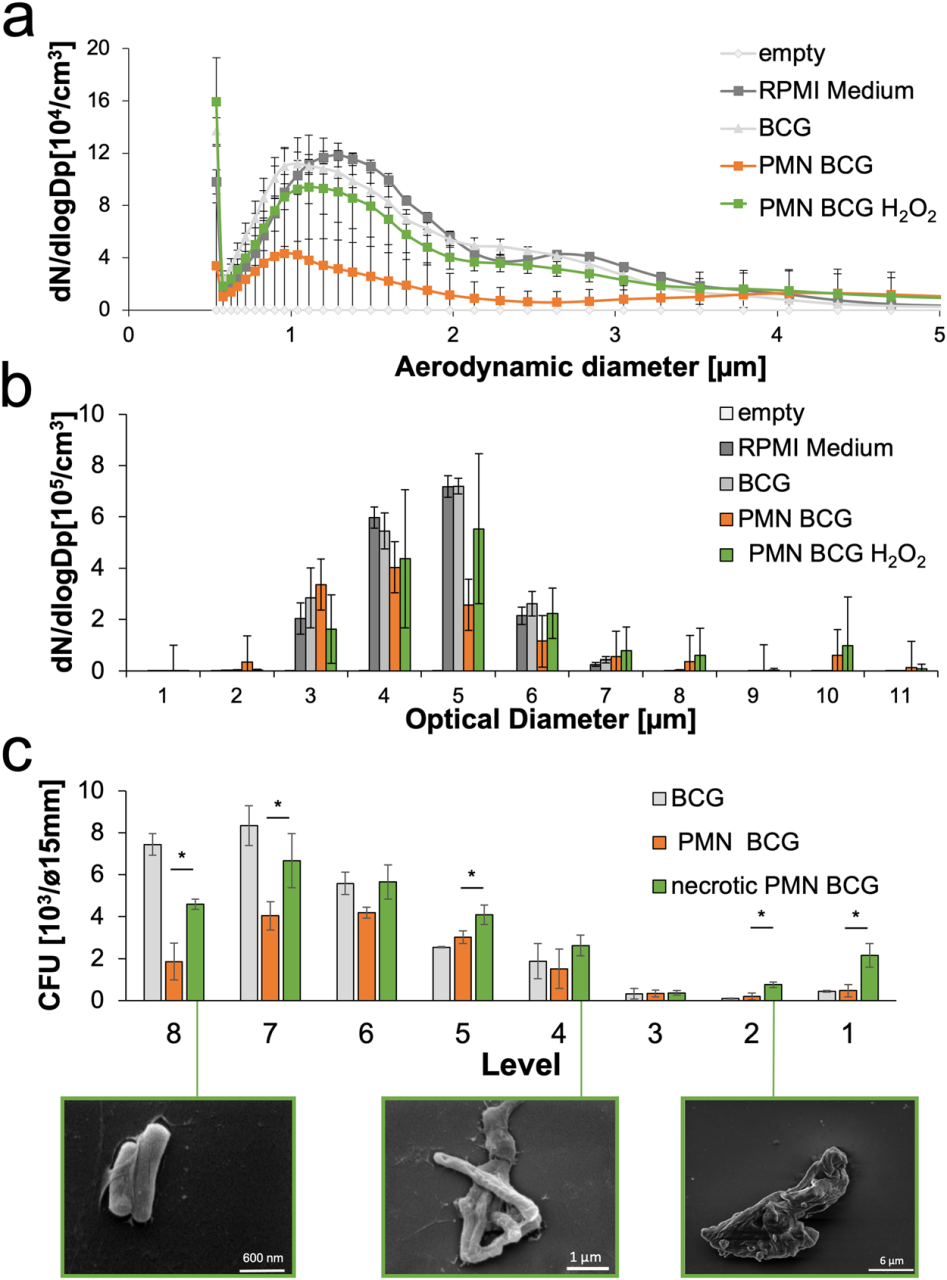
Aerosols from mycobacterium-infected neutrophils. (**a**) Frequency of aerosol particles with certain aerodynamic diameters upon ultrawave nebulization of free mycobacterial cells in RPMI, RPMI alone, and mycobacterium-infected but untreated or H_2_O_2_-treated necrotic neutrophils (n = 3). **(b)** Distribution of optical diameters of aerosol particles generated by ultrawave nebulization from a mycobacterial suspension in RPMI, RPMI alone, or mycobacterium-infected but untreated or H_2_O_2_-treated necrotic neutrophils (n = 3). **(c)** Mycobacterial viability was assessed by CFU in aerosols collected from different levels of an Andersen impinger after nebulization using the ultrawave nebulizer for either mycobacterial cells alone or mycobacterium-infected but untreated or H_2_O_2_-treated necrotic neutrophils. Selected fractions containing airborne mycobacteria from necrotic neutrophils (PMNs) were further analysed by SEM (below) (n = 3, *p > 0.001).

To analyse aerodynamic and optical diameter distributions of aerosol particles from either BCG-infected neutrophils or mycobacterial suspensions with or without H_2_O_2_ treatment, aerosols were generated by ultrawave nebulization. This nebulizer was suggested to be more appropriate for the generation of large aerosols^29^. Aerosol particle aerodynamic diameters, as analysed by APS, were similar independent of whether they were derived from free mycobacteria or infected intact or necrotic H_2_O_2_-treated neutrophils. Empty medium (RPMI) aerosols seemed to be slightly.

larger in their aerodynamic diameter but not in a statistically significant manner (p = 0.463). As observed before (Fig. 3) for PBS aerosols, empty RPMI aerosols were similar in size to those of free mycobacteria. Notably, reduced numbers of aerosol particles were generated from infected intact neutrophils (Fig. 6a). In contrast, the average optical diameters of aerosols generated from necrotic infected neutrophils, free mycobacteria or RPMI alone were larger than those from intact infected neutrophils but not in a statistically significant manner (p = 0.342) (Fig. 6b). The aerodynamic diameter correlated with the particle distribution determined by the recovery of CFU in the Andersen impinger. Notably, despite initial killing of the mycobacteria by H_2_O_2_ during induction of neutrophil necrosis, in comparison to the viability of BCG aerosol samples from non-treated neutrophils (see Fig. 5 for comparison), we observed more viable mycobacteria in aerosols from necrotic neutrophils than in those from intact BCG-infected neutrophils. Aerosols from necrotic mycobacterium-infected neutrophils contained a significantly increased number of viable mycobacteria on levels 1 and 2 of the Andersen impinger, corresponding to aerodynamic diameters between 6.5 and 8.0 µm. These aggregates were not observed in aerosol particles from intact mycobacterium-infected neutrophils or in those from free mycobacterial suspensions. Additionally, for aggregates of viable mycobacteria at levels 1 and 2, a shift in aerosol size distribution was observed at the lower impinger levels. While a great proportion of viable mycobacteria were found in aerosols from free BCG at levels 7 and 8, more viable mycobacteria were found in aerosols generated from infected H_2_O_2_-treated necrotic neutrophils at levels 6 and 7 (Fig. 6c). This result suggests an overall shift in the aerosol size proportion between free bacterial aerosols and necrotic neutrophil-derived mycobacterial aerosols. The compositions of mycobacterial aerosols generated from infected intact and necrotic neutrophils were analysed by SEM. Mycobacteria from infected but intact neutrophils primarily appeared as singlets and sometimes intact neutrophils (data not shown). In contrast, aerosols from H_2_O_2_-treated necrotic mycobacterium-infected neutrophils appeared as single bacterial cells or doublets at level 8, clumps at level 4, and aggregates associated with residual neutrophil material at level 1, which corresponded to those larger aggregates containing higher numbers of viable mycobacteria found on levels 1 and 2 (Fig. 6d).

Taken together, these results show that mycobacteria in aerosols quickly lose viability when airborne, likely due to dehydration. Induction of necrotic cell death in infected neutrophils generated mycobacterial aerosols with a tenacity similar to that of aerosols containing free mycobacteria but with larger multibacillary aggregates associated with neutrophil residues, which carried higher numbers of viable mycobacteria, suggesting protective shielding of mycobacteria from dehydration.

## Discussion

It is well established that *M. tuberculosis* can be transmitted through the air, but little is known about the biophysical properties of mycobacteria that allow successful transmission. Our study on *M. bovis* BCG revealed that the aerodynamic diameter as well as the mass density of mycobacterium-containing aerosols could allow long-distance transport, but airborne mycobacteria quickly lose their viability. Despite the reduction in mycobacterial viability during necrosis induction by H_2_O_2_, aerosolized mycobacteria from infected necrotic neutrophils maintained a higher viability as determined by CFU than those in aerosols derived from intact neutrophils. Enhanced aggregate formation and association with necrotic neutrophil material likely contributed to mycobacterial viability, probably through protecting the bacterial cells from desiccation. Our study on *M. bovis* BCG suggests that a major portion of living mycobacteria could be transmitted over short distances.

Airborne particles are exposed to different environmental factors, including varying RH. After the release of mycobacterium-containing aerosols from the respiratory tract, the bacteria are coated with water. During their airborne lifetime in air, water will condense on and evaporate from the bacterial surface, depending on the ambient RH. On short distance transmission (<1 m) of airborne bacteria, they will be coated with water, and the water layer will influence mainly the airborne behaviour. On long-distance transmission (>1 m), the ability of the bacterium to endure dehydration and the biophysical characteristics of the bacterium, such as mass density and hygroscopicity, will determine the success of airborne transmission.

### Short-distance transmission

Short-distance transmission is defined as transmission of pathogens over less than 1 m and thus facilitates transmission to close contacts^17^. The aerodynamic diameter of mycobacterium-containing aerosols was measured using an aerodynamic particle spectrometer (APS, Fig. 3). Since the aerodynamic diameter did not differ between empty and mycobacterium-containing aerosols, the aerodynamic diameter is likely defined by the surface tension of the buffer used, i.e., PBS. Interestingly, the optical diameter, which can be analysed simultaneously with the aerodynamic diameter, revealed a difference between empty and mycobacterium-containing aerosols upon jet nebulization. As mycobacteria are oval shaped, they can show a smaller aerodynamic than optical diameter because they can align along the air flow direction. Notably, this difference was not observed in aerosols generated by ultrawave nebulizing. The two nebulizers differed in their ability to generate viable BCG-containing aerosols. Despite comparable aerodynamic diameters of airborne BCG generated by both systems (Fig. 4b; and data not shown), the number of viable airborne BCG was reduced upon ultrawave aerosolization (Fig. S2).

Additionally, aerosols generated by the two types of nebulizers had different tenacities. Those produced by the ultrawave nebulizer fell out of air faster than those generated from jet nebulization despite both aerosol types showing comparable aerodynamic diameter distributions (Fig. 4b; and data not shown). Thus, aerosols generated by different forces behave differently. The different ways of expelling infectious aerosol particles, i.e., by coughing, sneezing, speaking or singing, may therefore drastically influence the particle behaviour, as reviewed by Gralton *et al*.^30^.

Most mycobacterium-containing aerosols consist of one or two bacterial cells and, rarely, a clump of bacteria (3–5 bacilli) with a layer of buffer around the bacterial nuclei (Fig. 2c). However, compared to single bacteria, multibacillary clumps were found to be more efficient in inducing necrotic cell death in macrophages, a prerequisite for promoting infection^31,32^. Aerosols ranging from 1–5 µm in size have been shown to be prone to reach the lower respiratory tract, even though deposition is less frequent for larger particles^20,22^. Our observation that settling velocities were similar between BCG aerosols consisting of single bacteria and clumps indicates that multibacillary aggregates can remain airborne for the same length of time as single bacteria (Fig. 4a). Thus, the small fraction of aerosolized mycobacterial clumps may still be as relevant as single bacteria for the initiation of infection^20,31,32^. Neutrophils represent the predominant mycobacterium-infected cell population in pulmonary samples from active TB patients, such as bronchoalveolar lavages or sputum, and they succumb to necrotic cell death upon infection with virulent *M. tuberculosis* strains^12,13,33^. To mimic aerosolization of mycobacteria from active TB patients upon coughing, induction of necrotic cell death in infected neutrophils promoted the formation of mycobacterial clumps associated with neutrophilic remnants (Fig. 6). Thus, mycobacterial clumps derived from necrotic neutrophils allow short-distance transmission of *M. tuberculosis* (Fig. 6). As we were interested in the effects of necrotic neutrophils on the composition of mycobacterium-containing aerosols, we excluded other interesting potential aerosol components, such as mucus, and other potential host cells, such as macrophages. However, one should be aware that they may also influence the tenacity of mycobacterium-containing aerosols. Animal aerosol infection studies using necrotic neutrophils will be required to confirm whether single bacteria differ from aggregates with respect to infectivity.

More importantly, airborne mycobacteria lost their viability after a short period of exposure to ambient air in the aerosol chamber. The death of airborne bacteria has been described as a two-step process^34^. In our experiments, most bacteria lost viability, as determined by CFU, within the first 30 min (Fig. 4c) after they became airborne, which was likely due to the sheering stresses during the aerosolization processes. Second-stage death affects bacteria that are airborne for longer time periods, for example, by nutrient deprivation and UV light^26,34,35^. As we continuously produced aerosols while we sampled the ambient air of our experimental box, we excluded second-stage death effects other than dehydration and nutrient deprivation that occur inside the Andersen impinger. One experimental study revealed that the viability of the wild-type *M*. *bovis* strain 61/4086/00 spoligotype 21 remained at 80% after nebulization^35^. These results are in striking contrast to our results using *M. bovis* BCG, which revealed a drop in viability by > 90% as a result of first-stage death upon aerosolization. Gannon *et al*. used a 135 l container to collect aerosols and a slow air drought of 14 l/min at a RH of 75%, which was in contrast to our set up of a 27 l box and an airflow of 28 l/min. Our findings, however, are comparable to those in studies of structurally unrelated gram-negative *E. coli* cells^36^. Thus, the faster air exchange of one complete ambient air volume per min could have reduced the viability of airborne mycobacteria. Furthermore, we used a commercial nebulizer for therapeutic medication, which generated aerosols by air drought, and the shear forces could additionally have affected mycobacterial viability. Other biological as well as experimental factors that may contribute to such differences are the mycobacterial strains used, the conditions of inoculum preparation, the ambient RH and the temperature during the experiment. However, in addition to those limitations, the small box allowed the workflow to be undertaken under a sterile work bench at BSL-2 conditions and will be also used in the future in a BSL-3 laboratory.

### Long-distance transmission

The success of airborne transmission depends on the mycobacterium’s ability to remain airborne as well as viable for prolonged time periods and cover distances of over one metre, which depends on different factors, including environmental cues, such as ambient humidity^17^. A relationship between the efficacy of airborne transmission of *M. tuberculosis* and relative environmental humidity has been described previously^4,27^. Higher transmission rates were observed at a higher RH. Thus, it was hypothesized that high humidity can protect mycobacteria against UV light and desiccation^26^. Experimental studies revealed that only humidities between 60 and 80% RH were supportive of transmission. At humidities >85% RH, the effect was reversed, and lower transmission rates were observed^4,26,27^. By measuring the volumes of single mycobacterial cells, the hygroscopicity of mycobacteria was shown to be saturated below 80% RH, (Fig. 1b) indicating that an excess of water in air cannot be further adsorbed by mycobacteria but is likely condensed around bacterial nuclei. It should be noted that mycobacterial cells quickly reacted to reduction in humidity, as they lost 32.7% of their mass when the RH was lowered from 90% to 0% (Fig. 1c). Interestingly, even though we observed such a drastic change in the mass and volume of the mycobacteria, in previous studies performed by Peccia *et al*. (2001), no change in the aerodynamic sizes of airborne bacteria was observed at different RHs ranging from 20% to 90%^37^. This result can also be seen in Fig. 4, where the measured aerodynamic diameter of aerosols did not vary even though the ambient air of the chamber lost humidity during the experiment. As nebulization inside the chamber increased the RH up to 90%, the ambient air of the chamber quickly returned to the room RH of 60–75% by air exchange after nebulization.

At a lower RH of 65%, mycobacteria became sensitive to dehydration, which reduced their viability 30 min after exposure to air by more than 30%. As water evaporates from the bacterial culture, the salt content increases in the sample, and therefore, bacterial dehydration occurs more rapidly. This phenomenon can be seen in the increased rate of viability loss after 60 min of exposure. However, since body fluids also contain salt, it is not inaccurate to measure the effects of dehydration in a salt solution.

However, the loss of viability in air occurred even quicker (Fig. 4), as bacteria were already stressed by the process of aerosolization (Fig. 2b). We may also underestimate the actual number of viable bacteria, as the bacteria were washed from the glass slide they were collected onto after sampling. When controlling this procedure, we already observed a loss of up to 10% of the viable mycobacteria (Fig. S3). The same effect was described for the release of aerosols from the respiratory tract, where high sheer forces are needed to release bacteria from cavities and bronchial obstruction and to overcome mucus viscosity^19,38^.

When environmental influences are excluded, Archimedes’ principle, which describes the interplay between gravity force on a particle as defined by its mass density and buoyancy depending on its volume, characterizes an aerosol particle’s properties. We determined the mass density of a single mycobacterium as 1.79 ± 0.08 g/cm^3^ at 65% RH (Fig. 1b). Therefore, we were able to calculate the settling velocity of mycobacterial particles using Stokes law, depending on their size and mass density. Notably, the difference in mass density between PBS (1 g/cm^3^) and a mycobacterial cell (1.79 ± 0.08 g/cm^3^) did not affect the aerosol velocity, especially when the size ranged between 1 and 5 μm, the range of mycobacterial aerosol sizes measured in our experiments. The distributions of aerodynamic diameters generated are dependent on the type of nebulizer used, which, however, in our hands, produced aerosol sizes that have been shown to reach the lower respiratory tract^6,7^.

Successful infection may therefore be determined by the distance a particle can travel through air and the time before viability and infectivity are reduced. Therefore, single mycobacteria are able to cover longer distances, whereas clumping preserves mycobacterial infectivity and increases virulence^26,31,32^. Therefore, clumping, though lowering the persistence in air, is beneficial for mycobacteria not only to infect alveolar macrophages but also to more quickly drive these cells into necrotic cell death and start the cycle of infection, growth and necrotic cell death, as described before^14,32^. The infectious dose for *M. tuberculosis* has been suggested to be rather low, i.e., less than 10 bacterial cells. As the aerosol velocity is not affected in the described size range, we cannot exclude the possibility of long-distance (>1 m) transmission of single mycobacteria. Computer simulation of airborne transmission, taking into consideration the different aerosol sizes determined here as well as the effects of air movement and particle transport, will help to solve the question of which aerosol compositions bear an increased risk of infection.

Our study provides insights into the biophysical properties of airborne mycobacterial transmission, indicating that transmission is more likely over a short time. Mimicking the situation in active TB patients, aerosols from mycobacterium-infected necrotic neutrophils showed enhanced clumping by association with neutrophil remnants, which can limit the transmission radius but, at the same time, increase infectivity upon inhalation. The quick loss of viability after exposure to ambient air likely reduces the chance of successful infection upon transmission over longer distances^39^. Association with neutrophils can prolong viability during air transmission likely by protection against dehydration and other environmental factors. Therefore, our data suggest that mycobacterial transmission can occur mainly between close contacts; for example, it can occur between household members and people in narrow occupational facilities as well as between closely interacting patients and health care workers.

## Methods and Materials

### Bacterial strains and growth

The *M. tuberculosis* strain Beijing 49/02 and the *M. bovis* BCG strain Pasteur (BCG) were used. Mycobacteria were grown in Middlebrook 7H9 broth (BD Bioscience, Franklin Lakes, New Jersey), 0.05% Tween 80 (Serva, Heidelberg, Germany) and 10% OADC (BD Bioscience, Franklin Lakes, New Jersey).

### Viability of mycobacteria

To assess the concentration of viable mycobacteria, colony-forming units (CFU) were determined by plating BCG-containing solutions after separating bacteria with a cannula (∅ 0.9 mm, B. Braun, Melsungen, Germany) on sterile 7H11 agar plates. Colonies were counted after 4 weeks of culture at 37 °C. Additionally, the ratio between live and dead bacteria was analysed using a LIVE/DEAD™ BacLight™ Bacterial Viability Kit (ThermoFisher Scientific, Waltham, Massachusetts). For that, airborne bacteria were sampled on glass and stained according to the manufacturer’s instructions. Slides were observed using an inverted Zeiss LSM 510 fluorescence microscope (63x objective; Zeiss, Oberkochen, Germany). Images were analysed by Zeiss LSM Image Browser software.

### Mass density

The following formula was used to calculate the mass density of a single bacterium: mass density = volume of bacterial cell / weight of bacterial cell. The volume of one bacterium was determined using atomic force microscopy (AFM, Oxford Instruments, Asylum Research, Santa Barbara, California; Cantilever OMCL-AC160TS, Olympus, Tokyo, Japan) at 65% RH. The weight of one bacterium was determined by defining the number of single bacteria in culture samples using a sperm counting chamber (Marienfeld, Lauda Königshofen, Germany), weighing the BCG culture after drying and adjusting to 65% RH. This approach allows the calculation of the weight of one bacterial cell (weight of total bacteria/number of bacteria).

### Hygroscopicity

Hygroscopicity is defined as the ability to absorb water from ambient air. The amount of available water is dependent on the RH. BCG in PBS (Sigma-Aldrich, St. Louis, Missouri) was deposited on coverslips by centrifugation (800 × g for 10 min). During centrifugation, the PBS was removed by filters; thus, the bacteria were dried. The volume of a single BCG particle was determined by AFM under different RHs. The RH was adjusted by deliquescence salts inside a closed fluid chamber using saturated salt solutions of potassium chloride for 85%, sodium nitrate for 65% and lithium chloride for 20% RH (ThermoFisher Scientific, Waltham, Massachusetts).

### Dynamic vapour sorption (DVS)

Sorption behaviour was characterized utilizing a DVS-1 (Surface Measurement Systems, London, UK). Dynamic vapour sorption (DVS) is a gravimetric technique that measures how quickly and how much of a solvent, typically water, is absorbed or desorbed by a sample. By varying the vapor concentration in the sample’s environment, the changes in mass can be monitored. The data were used to calculate sample characteristics such as sorption isotherms, hygroscopicity or water uptake at a given humidity.

Approximately 2.6 mg of the bacteria were transferred directly from a storage vessel into an aluminium DVS sample holder. The sample holder was then put into the measuring chamber, which was set at 90% RH. The measurement consisted of two steps. After reaching mass consistency (mass change <0.005%/min), the RH was decreased from 90 to 0%. The measurement was completed when mass consistency was again achieved.

### Production and analysis of *in vitro*-produced aerosols

**Figure 7:**
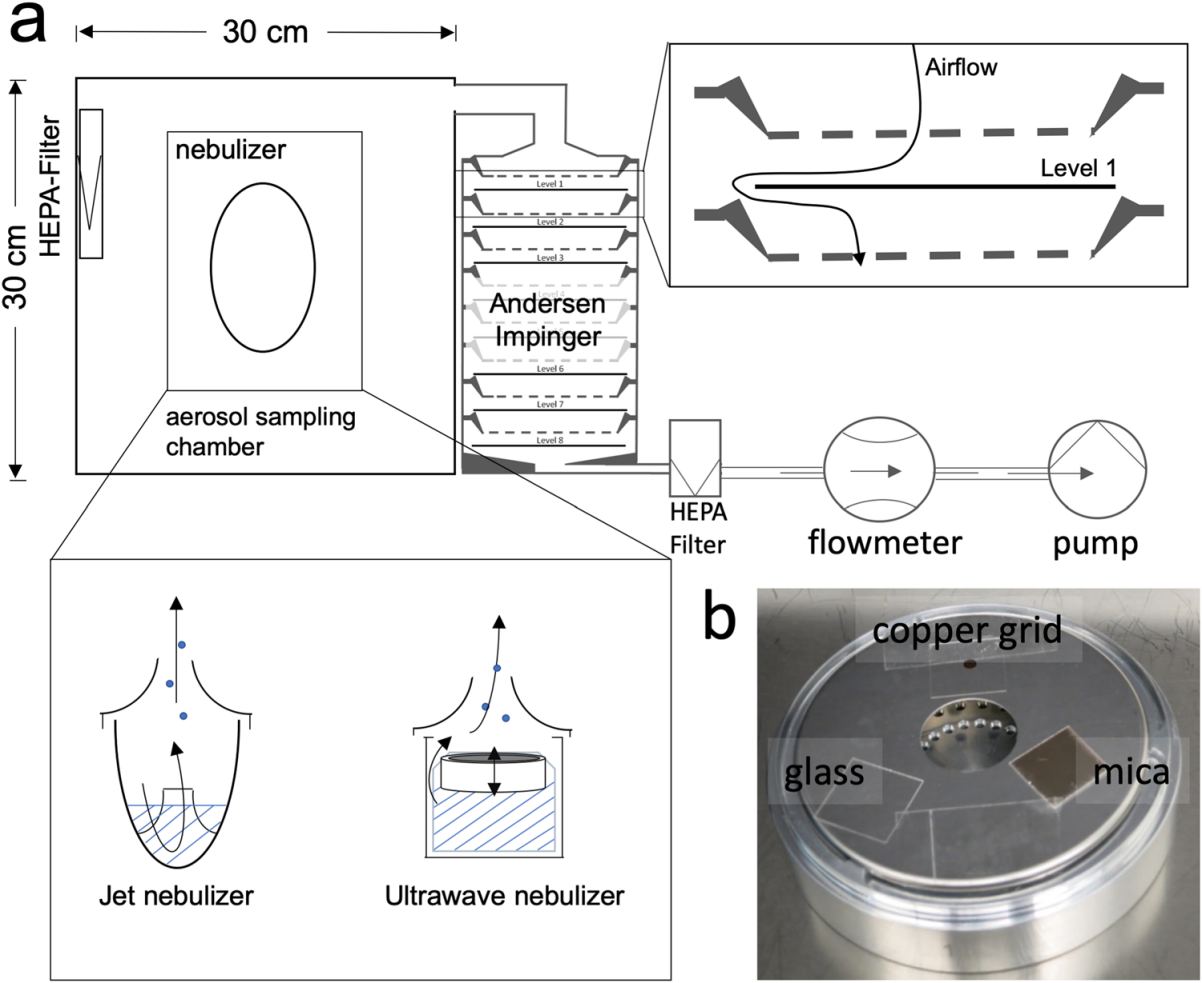
Schematic overview of the aerosol collection device (ACD). (**a**) The device consists of an acrylic glass chamber of 27 l, including a HEPA filter to remove particles from incoming air. The ACD is connected to nebulizers, which use either jets or ultrawaves to generate aerosols. The air containing these aerosols is actively pumped through the multi-stage impinger. The airflow is measured by a flowmetre. The HEPA filter in front of the pump and flowmetre protects the equipment. **(b)** A photograph of level 1 of the impinger equipped with three different surfaces to collect settling aerosols, i.e., (I) glass coverslip for viability testing as appraised with CFU and live/dead microscopy staining, (II) copper grid for EM analysis, and (III) mica for atomic force microscopy analysis.

To assess the viability of mycobacterial aerosol particles by CFU, EM and live/dead staining, we constructed an aerosol-collecting device (ACD) (Fig. 7). Mycobacteria were nebulized from PBS suspensions, generating aerosol droplets with bacterial nuclei (“mycobacterium-containing aerosols”) and without (“empty PBS aerosols”).

The home-built aerosol collecting device (ACD) contained a small clean box (27 l) connected to an Andersen cascade impinger (Copley Scientific, Nottingham, UK). Intake air flow was cleaned by a HEPA filter (SF-HA 50 HEPA AirClean, Miele, Gütersloh, Germany). All experiments using the ACD were performed under a sterile class II working bench. Aerosols were expelled into the small clean box using two different nebulizers. The jet nebulizer (IH18, Beurer, Ulm, Germany) uses compressed air flow with a high velocity to produce aerosols, whereas the ultrawave nebulizer (U22 Microair, Omron, Kyoto, Japan) produces aerosols by an oscillatory membrane (Fig. 7a). Aerosols were expelled for the whole sampling time of 10 min, and aerosols were sampled during nebulization. From the clean box, ambient air was pumped either through an Andersen cascade impinger (ACI) consisting of 8 levels (−2 to 5), collecting aerosols at 28 l/min, or an aerodynamic particle spectrometer (APS 3221, TSI, Shoreview, Minnesota). The APS provides information about the aerodynamic and optical diameters of airborne particles as dN/dlogDp values. dN (or ΔN) is the number of particles in a certain range channel (total concentration of particles), and dlogDp (or ΔlogDp) is the difference in the log of the channel width.

On each level of the ACI, different sampling devices were placed for particle analysis (Fig. 7b). To test mycobacterial viability, aerosol particles were collected on glass and either washed with 500 µl of PBS for CFU or incubated immediately after collection with LIVE/DEAD stain. For correlative transmission electron microscopy, aerosols were collected for 10 min at 28 l/min on carbon-coated, sputtered 500 mesh copper grids (Electron Microscopy Sciences (EMS), Hatfield, Pennsylvania). Air was pumped by a vacuum pump (Copley Scientific, Nottingham, UK) and controlled by an airflow metre (Copley Scientific, Nottingham, UK). Ambient ACD air was filtered by a HEPA filter before entering the pump (Patronenfilter- HS- Mikroseal JG-S, HS-Luftfilterbau GmbH, Kiel, Germany).

### Negative stains

For each level of the Andersen impinger, 3 carbon-coated, sputtered 500 mesh copper grids (Electron Microscopy Sciences (EMS), Hatfield, Pennsylvania) were provided. Subsequently, after sampling, grids were stained for 2 min with 2% uranyl acetate (EMS, Hatfield, Pennsylvania) and analysed under a transmission electron microscope (Philips CM120/Gatan MSC 794, Hillsboro, Oregon). For each grid, 100 bacteria were analysed, but not all were documented. The results are shown as number out of 100.

### Scanning electron microscopy

Samples were fixed on ice with 2.5% glutaraldehyde (EMS, Hatfield, Pennsylvania) in PBS (Sigma-Aldrich, St. Louis, Missouri), stained with 2% Osmeon (EMS, Hatfield, Pennsylvania) in PBS on ice (Sigma-Aldrich, St. Louis, Missouri) and washed with ddH_2_O. Samples were dehydrated in ethanol solutions of 30%, 50%, 70%, 90% and three times 100% for 3 min each (Carlroth, Karlsruhe, Germany). For hexamethyldisilazane (HDMS, Sigma-Aldrich, St. Louis, Missouri) drying, samples were immersed for 3 min in 50% HMDS in ethanol and 3 min in 100% HMDS and dried. Dried samples were mounted on stubs and sputter coated with 10 nm gold (Q150R Quorum Technologies, Lewes, GB). Samples were examined with a Philips XL30ESEM/Point Electronic DISS5 (Philips, Hillsboro, Oregon).

### Ethical approval

All experiments were performed in accordance with relevant guidelines and regulations. Human neutrophils were isolated from healthy volunteers over the age of 18 upon informed consent obtained from all donors approving and authorizing the use of the blood. The protocol and procedure were approved by the Ethical Committee of the University Lübeck, Germany (https://www.uni-luebeck.de/forschung/kommissionen/ethikkommission.html),under Ethical Approval File No. 22–202 A.

### Isolation, culture and infection of neutrophils

Human neutrophils were isolated from heparinized peripheral blood from healthy donors. Neutrophils were separated by centrifugation by first using a Histoplaque 1199 (Sigma-Aldrich, St. Louis, Missouri) gradient and second using a Percoll gradient (Sigma-Aldrich, St. Louis, Missouri), as described previously^14^. Neutrophils were cultured in RPMI medium (Invitrogen, Carlsbad, California) supplemented with 10% FCS (Sigma-Aldrich, St. Louis, Missouri) and 2 mM glutamine (Invitrogen, Carlsbad, California) and infected at an MOI of 1. BCG was opsonized with autologous serum from healthy donors and incubated for 30 min at 37 °C. Infected neutrophils were incubated for 36 h at 37 °C/5% CO_2_. In some experiments, neutrophils were treated with 0.3% H_2_O_2_ (Omnilabs, Hamburg, Germany) to induce necrosis.

### Necrotic cell death induction and lactate dehydrogenase assay

When using BCG as a *M. tuberculosis* surrogate, artificial necrosis induction required the addition of an ROI donor to the cell cultures. Treatment of BCG-infected human neutrophils by hydrogen peroxide (H_2_O_2_) robustly initiated necrotic cell death, as determined by measuring the activity of lactate dehydrogenase (LDH) in neutrophil cultures; LDH is a strictly cytoplasmic enzyme that is detected in supernatant after plasma membrane rupture.

To experimentally drive infected neutrophils into necrotic cell death, cells were treated with 0.3% H_2_O_2_. The 100% reference was determined by lysing neutrophils with the detergent Triton X-100 (0.1% in PBS, Omnilabs, Hamburg, Germany). For measuring necrotic cell death, neutrophil culture supernatants or total cell lysates were transferred to 96-well plates and serially diluted 1:2. LDH activity was measured using a mixture of diaphorase/NAD + and iodotetrazolium chloride/sodium lactate following the manufacturer’s instructions (Roche, Mannheim, Germany). Samples were analysed by measurement of optical density using an Opsys MR ELISA reader (Dynex Technology, Chantilly, Virginia) at a 490 nm wavelength.

### Statistical analyses

All data analysis and statistics were performed using Excel. Data were entered into a standard table, with one row representing one sample. Statistical analysis was performed by determining the average and standard deviation and analysis of variance (ANOVA), and all significant differences are reported at 95% confidence intervals. Significant differences with p-values <0.01 are represented by one star, two stars represent p-values <0.001, and three stars represent p-values <0.0001.

### Statement of ethical approval

The authors state that human neutrophils were isolated from healthy donors (18 years of age or older) upon written consent approving and authorizing the use of the blood. All methods were carried out in accordance with relevant guidelines and regulations, and all experimental protocols were approved by the Ethical Committee of the University Lübeck, Germany (File No. 22–202 A).

## Supplementary information

Figure S1, Figure S2, Figure S3.

## Data Availability

All datasets generated during and/or analysed during the current study are available from the corresponding author on request.
