# Author Correction: Enhanced tenacity of mycobacterial aerosols from necrotic neutrophils

**DOI:** 10.1038/s41598-020-70097-9

**Published:** 2020-08-04

**Authors:** E. Pfrommer, C. Dreier, G. Gabriel, T. Dallenga, R. Reimer, K. Schepanski, R. Scherließ, U. E. Schaible, T. Gutsmann

**Affiliations:** 1 Heinrich Pette Institute, Leibniz Institute for Experimental Virology, 20251 Hamburg, Germany; 2 Forschungszentrum Borstel - Leibniz Lung Center, 23845 Borstel, Germany; 3 Leibniz Institute for Tropospheric Research, 04318 Leipzig, Germany; 4 Leibniz Research Alliance INFECTIONS’21, Borstel, 23845 Germany; 5 German Center for Infection Research (DZIF), Partner Site Hamburg-Lübeck-Borstel, Germany; 6 Christian Albrechts University of Kiel, 24118 Kiel, Germany

Correction to: *Scientific Reports*https://doi.org/10.1038/s41598-020-65781-9, published online 08 June 2020

The original version of this Article contained errors within the affiliations section.

Affiliation 4 was incorrectly given as ‘Leibniz Research Alliance INFECTIONS’21, Leipzig, Germany’. The correct affiliation is listed below:

Leibniz Research Alliance INFECTIONS’21, Borstel, 23845, Germany

Also, Affiliation 5 was incorrectly given as ‘German Center for Infection Research, TTU-TB, Borstel, 23845, Germany’. The correct affiliation is listed below:

German Center for Infection Research (DZIF), Partner Site Hamburg-Lübeck-Borstel, Germany.

Finally, the original HTML version of this Article omitted an affiliation for G. Gabriel. The correct affiliations for G. Gabriel are listed below:

Heinrich Pette Institute, Leibniz Institute for Experimental Virology, Hamburg, 20251, Germany.

Leibniz Research Alliance INFECTIONS’21, Borstel, 23845, Germany.

German Center for Infection Research (DZIF), Partner Site Hamburg-Lübeck-Borstel, Germany.

These errors have now been corrected in the PDF and HTML versions of the Article.

