# Supplementary material for: Enhanced tenacity of mycobacterial aerosols from necrotic neutrophils
Source: Sci Rep. 2020 Jun 8;10:9159. doi: 10.1038/s41598-020-65781-9 (PMC7280268; doi:10.1038/s41598-020-65781-9)
Supplement: Supplementary file 1 — Figure S1, Figure S2, Figure S3. [file 41598_2020_65781_MOESM1_ESM.docx]

**Enhanced tenacity of mycobacterial aerosols from necrotic neutrophils**

***Supplemental Material***

E. Pfrommer^1,2,3,4^, C. Dreier^1,4^, G. Gabriel^1,4,5^, T. Dallenga^2,5^, R. Reimer^1^, K. Schepanski^3,4^, R. Scherließ^6^, U. E. Schaible^2,4,5*^, T. Gutsmann^2,4^

^1^Heinrich Pette Institute, Leibniz Institute for Experimental Virology Hamburg, 20251, Germany

^2^Forschungszentrum Borstel - Leibniz Lung Center, Borstel, 23845, Germany

^3^Leibniz Institute for Tropospheric Research, Leipzig, 04318, Germany

^4^Leibniz Research Alliance INFECTIONS’21, Borstel, 23845, Germany

^5^German Center for Infection Research (DZIF), Partner site Hamburg-Lübeck-Borstel, Germany

^6^Christian Albrechts University of Kiel, 24118 Kiel, Germany

* Corresponding author: uschaible@fz-borstel.de

**Supplementary Figures**


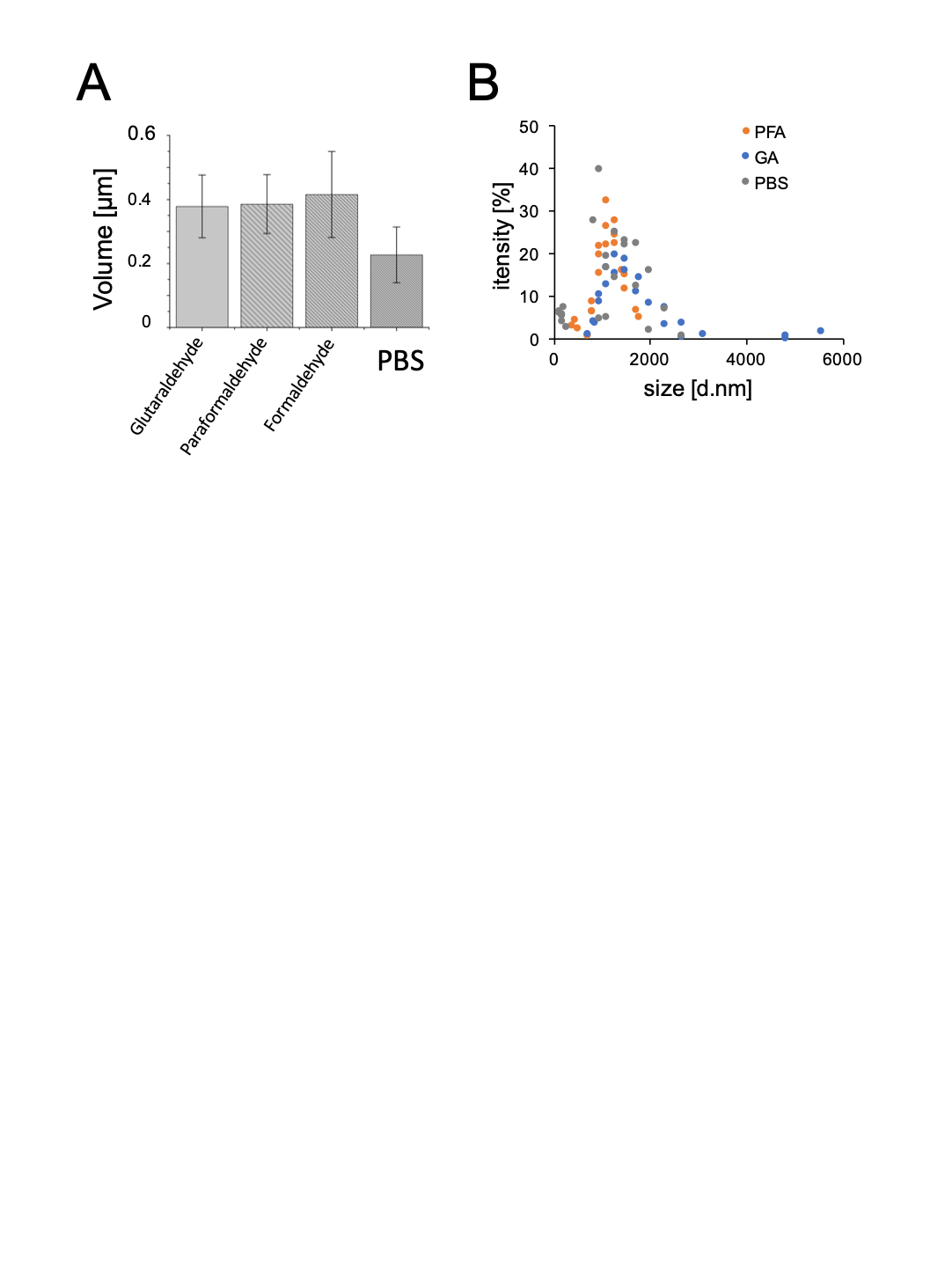


**Fig. S1 Effect of aldehyde fixation on mycobacteria (A)** Averaged volume of single mycobacterial cells as determined by AFM upon inactivation for 30 min with different aldehyde fixatives or left viable (non-contact mode, AC165TS cantilever). All aldehydes increased the volume of the mycobacteria compared to native particles (n=20). **(B)** Analysis of either paraformaldehyde (PFA) or glutaraldehyde (GA) treated, or non-treated (PBS) mycobacteria in solution using dynamic light scattering (n=3).

**Fig. S2 Comparison of BCG viability upon arosolization by jet vs. ultrawave nebulizer.** CFU determined from aerosolized BCG collected at the different Andersen Impinger levels, when either produced by jet or ultrawave nebulizer (n=1).

**Fig. S3 Impact of the washing step from the glass slides upon collecting BCG aerosols by Andersen Impinger.** A BCG solution with a known CFU was applied on to a glass slide and then removed by washing with PBS to measure the efficiency of the washing step. Through the washing, around 10 % of the viable BCG as measured by CFU were lost (n=1).
